# The effect of supervision on community health workers’ effectiveness with households in rural South Africa: A cluster randomized controlled trial

**DOI:** 10.1371/journal.pmed.1004170

**Published:** 2023-03-02

**Authors:** Mary Jane Rotheram-Borus, Karl W. le Roux, Peter Norwood, Linnea Stansert Katzen, Andre Snyman, Ingrid le Roux, Elaine Dippenaar, Mark Tomlinson

**Affiliations:** 1 Dept. of Psychiatry and Biobehavioral Sciences, Semel Institute, University of CA, Los Angeles, California, United States of America; 2 Institute for Life Course Health Research, Dept. of Global Health, Faculty of Medicine and Health Sciences, Stellenbosch University, Tygerberg, South Africa; 3 Dept. of Family Medicine, Walter Sisulu University, Mthatha, South Africa; 4 Primary Health Care Directorate, Old Main Building, Groote Schuur Hospital, Cape Town, South Africa; 5 Zithulele Training and Research Centre, Zithulele Hospital, Mqanduli District, Eastern Cape, South Africa; 6 Philani Maternal, Child Health and Nutrition Trust, Khayelitsha, Cape Town, South Africa; 7 School of Nursing and Midwifery, Queens University, Belfast, United Kingdom; Washington University in St Louis School of Medicine, UNITED STATES

## Abstract

**Background:**

Community health workers (CHWs) can supplement professional medical providers, especially in rural settings where resources are particularly scarce. Yet, outcomes of studies evaluating CHWs effectiveness have been highly variable and lack impact when scaled nationally. This study examines if child and maternal outcomes are better when existing government CHWs, who are perinatal home visitors, receive ongoing enhanced supervision and monitoring, compared to standard care.

**Methods and findings:**

A cluster randomized controlled effectiveness trial was conducted comparing outcomes over 2 years when different supervision and support are provided. Primary health clinics were randomized by clinic to receive monitoring and supervision from either (1) existing supervisors (Standard Care (SC); *n* = 4 clinics, 23 CHWs, 392 mothers); or (2) supervisors from a nongovernmental organization that provided enhanced monitoring and supervision (Accountable Care [AC]; *n =* 4 clinic areas, 20 CHWs, 423 mothers). Assessments were conducted during pregnancy and at 3, 6, 15, and 24 months post-birth with high retention rates (76% to 86%). The primary outcome was the number of statistically significant intervention effects among 13 outcomes of interest; this approach allowed us to evaluate the intervention holistically while accounting for correlation among the 13 outcomes and considering multiple comparisons.

The observed benefits were not statistically significant and did not show the AC’s efficacy over the SC. Only the antiretroviral (ARV) adherence effect met the significance threshold established a priori (SC mean 2.3, AC mean 2.9, *p* < 0.025; 95% CI = [0.157, 1.576]). However, for 11 of the 13 outcomes, we observed an improvement in the AC compared to the SC. While the observed outcomes were not statistically significant, benefits were observed for 4 outcomes: increasing breastfeeding for 6 months, reducing malnutrition, increasing ARV adherence, and improving developmental milestones. The major study limitation was utilizing existing CHWs and being limited to a sample of 8 clinics. There were no major study-related adverse events.

**Conclusions:**

Supervision and monitoring were insufficient to improve CHWs’ impact on maternal and child outcomes. Alternative strategies for staff recruitment and narrowing the intervention outcomes to the specific local community problems are needed for consistently high impact.

**Trial registration:**

Clinicaltrials.gov, NCT02957799.

## Abstract 2

In a randomised controlled trial, Dr. Mary Jane Rotheram-Borus and colleagues investigate the effect of supervision on community health workers’ effectiveness in households of rural South Africa.

## Author summary

**Why was this study done?:**

- There are too few health professionals in low- and middle-income countries, and there will be too few until about 2050.
- Healthcare tasks are being shifted to 2 million community health workers (CHWs).
- Yet, the efficacy of these CHWs often disappears when randomized controlled trials (RCTs) are ended and national programs are implemented.

**What did the researchers do and find?:**

- Existing government-employed CHWs serving 8 deeply rural health clinics were randomized by clinic in a cluster effectiveness RCT to either standard supervision or enhanced accountable monitoring and supervision by a nongovernment organization.
- The CHWs and perinatal mothers/children were monitored at 5 points over the first 2 years of life.
- Supervision did not lead to significantly better outcomes for mothers and children.
- Yet, it is noteworthy that 11 of 13 outcomes were better when CHWs received enhanced, accountable supervision, compared to standard care.

**What do these findings mean?:**

- A key component of implementation at scale in the real world is the ability to hold CHWs accountable and to release CHWs not meeting expectations.
- To successfully deploy CHWs in a manner that has significant and sustained improvements, the procedures for selecting and recruiting CHWs may be critical.

## Background

Community health workers (CHWs) are a key solution to meeting the health needs of billions of persons lacking sufficient access to professional healthcare workers. Tasks are being shifted from professional health workers to paraprofessional CHWs globally [1]. Yet, evaluations of CHWs’ impact have been mixed [2,3]. There are a relatively large number of efficacy trials that find benefits associated with CHWs [3–5]. However, moving from efficacy to effectiveness and then scale-up are complex processes and fraught with difficulties. In many cases, when these programs are scaled to larger populations, the effects evaporate [5]. Similarly, the evaluations of national programs have also found mixed results [6,7].

There are many reasons for these failures. Most randomized controlled trials (RCTs) devote considerable resources to staff selection/recruitment, training, and fidelity monitoring over time of manualized programs that target a single outcome. When scaled, most large public health systems fail to allocate sufficient resources to implement and manage these programs. Implementation often lacks fidelity [8], and managers lack the experience or skills to take complex interventions to scale [9]. An emerging area of consideration is the extent to which essential implementation features, such as thorough recruitment, supportive supervision, and quality management, are not replicated when taken to scale [10,11]. These components are rarely seen as integral to success as compared to the informational content and the manualised nature of the intervention [2].

It is likely that the potential of CHWs to impact outcomes is contingent on the workforce strategies for recruiting, training, monitoring, and supportive supervision. Our underlying hypothesis is that if the potential impact of CHWs programs is to be realized, understanding the contribution of a well-trained and well-supervised workforce is essential.

There have been few studies examining supervision, especially in low- and middle-income countries (LMICs). In 2017, Ballard and Montgomery [11] attempted to evaluate the efficacy of implementation strategies to improve CHWs outcomes. While they identified 14 interventions from 8,082 potential studies, the quality of the 14 reports was so inconsistent that a meta-analysis could not be conducted. Westgate and colleagues’ recent review [10] also relied only on case studies of large CHWs programmes, with few data, for example, informing supervision policies. The reviewers concluded that there was an urgent need for rigorous evaluations of how to best improve CHWs’ outcomes. This study fills this gap.

South Africa has 55,000 CHWs [12], and these CHWs are seen as a key component of the government’s plans to “Re-engineer Primary Health Care” [13–16]. In 2011, more than 33,000 of the 55,000 CHWs were reassigned from being based mostly at clinics to performing home visits [17,18]. Preliminary qualitative and observational studies have found CHWs embracing their redeployment [14] and that nurse supervisors based in clinics were better than nurses in community-based sites [19].

CHWs’ job is complex. Pregnant South African mothers face multiple interrelated challenges. South Africa is the country with the most HIV infections [20], about 30% of pregnant women are mothers living with HIV (MLH). South Africa also has the highest per capita ingestion of alcohol (even though about 50% do not drink alcohol), [21] as well as high rates of interpersonal violence [22]. Antenatal and postnatal depression are common [23]. Concurrently, their children are at risk of low birth weight [24] and suboptimal growth, often experiencing food insecurity [25]. Mothers are challenged to maintain their children’s health regimens, such as regular immunizations, getting children tested and protected from HIV, and ensuring that children have the attention needed to successfully complete their developmental milestones [26]. A CHW’s role is to help mothers with each of these tasks, protecting both her own health, as well as her baby’s. These challenges require CHWs who are generalists.

This study examines if supportive supervision of CHWs increases the impact of the existing government-employed CHWs workforce on the health outcomes of the mothers and children—examined in a cluster RCT. Given the structure of healthcare settings, we can only randomize CHWs by clinics, rather than individually assigning CHWs to receive supervision or not. In addition, because CHWs address multiple health challenges, our analytic strategy must concurrently examine a range of outcomes that shift over time. Improving one outcome (for example, increasing breastfeeding) is unlikely to positively impact the child’s completion of immunizations over time. We need an analytic strategy that allows us to analyze multiple outcomes that does not rely on a power-killing, Bonferroni-type correction. The generalist intervention requires that there be multiple significant outcomes to demonstrate an efficacious intervention and protect against Type 1 error. Our proposed strategy [27] addresses this challenge and also accounts for the correlation among the outcomes. With this analytic strategy, we can evaluate our hypothesis that supportive supervision is superior to standard care.

## Methods

### Study setting and participants

The study took place in the deeply rural King Sabata Dalindyebo Health Subdistrict of the OR Tambo District, Eastern Cape of South Africa [28]. The catchment area was served by 8 primary care clinics that refer patients to Zithulele District Hospital, a state-run facility that serves about 130,000 people [29]. The district is situated in the former Transkei homeland and is one of the poorest and most underdeveloped in the country, ranking below national standards in terms of access to water, sanitation, and healthcare [30]. Only 27% of households have access to communal taps, with 48% relying on unsafe river water, while 93% of households receive a government grant [30].

Prior to randomization, we documented the size of the clinics’ catchment areas, density of housing in the catchment area, the number of women initiating antenatal care at the clinic over the previous year, and the topography of the clinic catchment area (including the average slope). Due to substantial variations between some of the clinics’ catchment areas and size of their catchment populations, UCLA paired 2 sets of 4 clinics each to help create catchment areas that were largely similar for each pair and then used a random number generator to randomly assign one set of the matched pairs to either the Accountable Care (AC) or to the Standard Care (SC) conditions.

### Data collectors

Local women who lived in adjoining neighborhoods were recruited and trained as data collectors. These isiXhosa-speaking women also spoke English, typically had a 12th grade education, and were selected based on their good social skills, the ability to engage peers, and the responsibility and ability to complete interviews on a digital platform. Data collectors were assigned to clinics during the recruitment period and then conducted all assessments in the participants’ home or outdoors if there was no privacy in the home. Data collectors were blind to intervention condition but may have realized intervention status over time, based on mothers’ answers to assessment questions.

### Sample

All pregnant women over the age of 14 years, presenting to any of the 8 study clinics for antenatal care from June 2017 to September 2018, were invited to participate in the study and signed voluntary informed consent. Mothers’ consent was to participate in a study of maternal and child well-being, and mothers were blind to the supervision status of the CHWs routinely assigned to all pregnant and new mothers. Data collection at follow-up interviews continued until February 2021. COVID-19 led to a break in field operations with no in-home data collection from March to October 2020. During this period, telephone interviews were allowed from July to October 2020. Telephone interviews did not allow anthropomorphic measures to be gathered for 89/1,369 (6.5%) mothers or children on their follow-up interviews.

**Fig 1:**
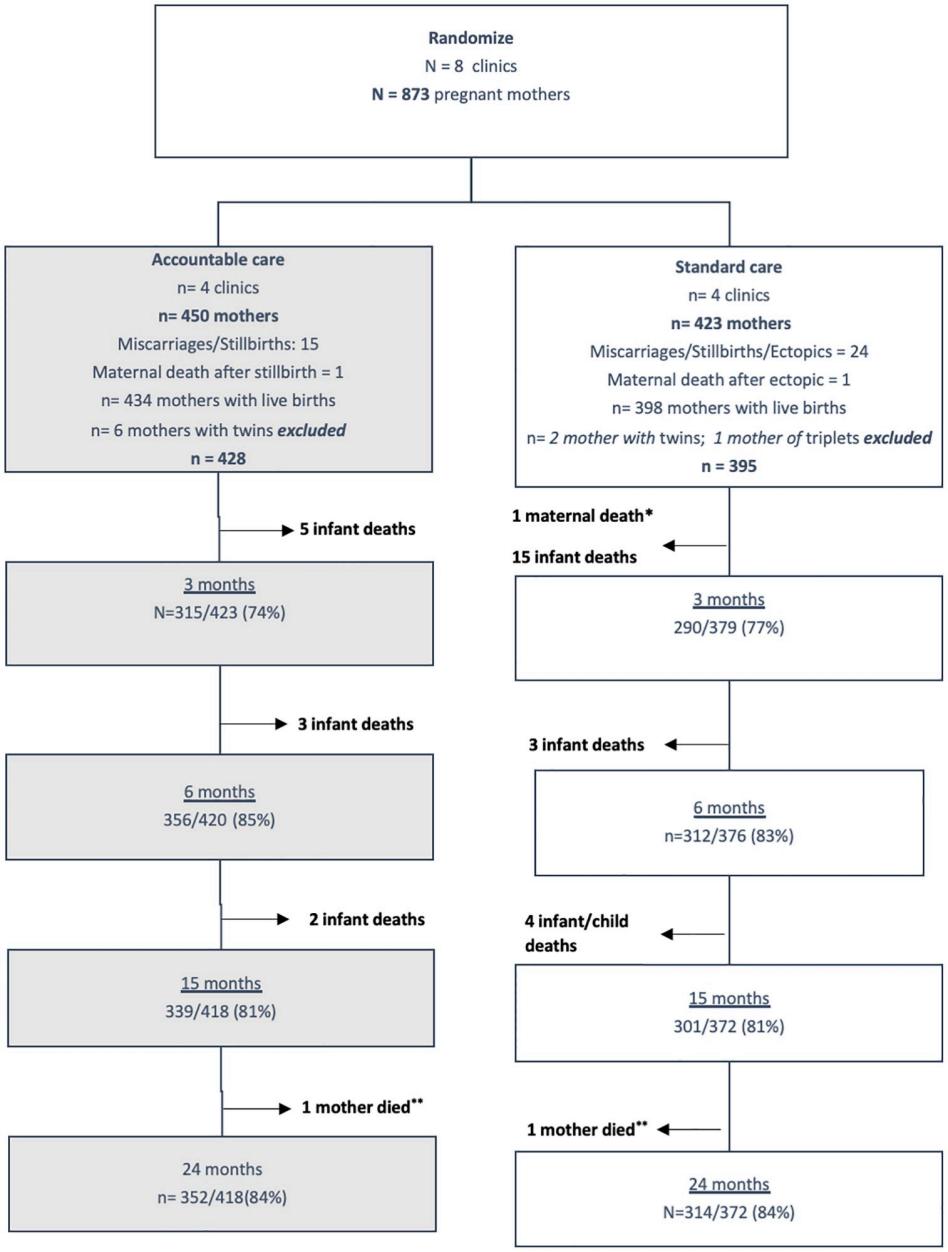
The flow of participants throughout the course of the trial. * Baby also died soon after birth, **Though mother died, follow up of child with caregiver was still performed, therefore denominator was not affected.

Pregnant women under the age of 18 years provided both youth assent and parent/guardian consent. Women with intellectual impairment or who demonstrated significant psychiatric problems at initial contact were excluded based on the interviewer’s evaluation. Data collectors reviewed all clinic and hospital antenatal and birth records to identify pregnant women and ensure that a consecutive series of 900 pregnant women were recruited; 27/900 women were then found ineligible (underage, not pregnant, recruited twice), resulting in 873 pregnant women. Twins/triplets were excluded because the developmental trajectories of these children have been found to be quite different in previous studies [31]. Given multiple births, miscarriages, still births, and maternal deaths, 50 participants (22/450 in AC; 28/423 in SC) were also excluded prior to the child’s live births. Fig 1 outlines the flow of participants throughout the study. The baseline assessment was conducted during pregnancy, and mothers and their babies were then reassessed at 3, 6, 15, and 24 months post-birth with follow-up rates of 75.5% (605/802; 22 deaths), 84% (669/796; 6 deaths), 81% (640/790; 6 deaths), and 84.2% (666/790), respectively. Only 9/873 participants were not reassessed at least once after the baseline assessment.

All interviews were recorded, and a random sample of about 10% of the 3,178 recorded interviews were reviewed for quality by an isiXhosa-speaking researcher. Collected data were reviewed weekly so that errors were queried and corrected.

#### Measures

*Background characteristics* collected included maternal age, whether in school currently, number of years of schooling completed, current employment, and the presence of a live-in partner or husband. Economic resources were identified as present (1) or absent (0) including food insecurity, monthly income (>2,000 South African Rand [ZAR]), access to electricity, and access to safe water. The number of adult and child household members were reported, food insecurity, receipt of the child support grant prior to childbirth for other household members), the number of previous births, HIV status, histories of lifetime suicide attempts, having a chronic illness, lifetime and recent number of sexual partners, and interpersonal violence. We also assessed alcohol use prior to recognizing one is pregnant and during pregnancy post-pregnancy discovery, and problematic alcohol use with the Alcohol Use Disorders Identification Test-C (AUDIT-C) [32] for the 2 time frames. The AUDIT-C is a 3-item scale indicating alcohol consumed in the last year; number of drinks daily when drinking; and number of times having more than 6 drinks in a day. A score >3 is considered problematic alcohol use.

*Reports of home visits*. Mothers reported visits by CHWs at each assessment.

#### Outcome measures

*Alcohol Use During Pregnancy*. Mothers were asked if they ever used alcohol after discovering they were pregnant at the baseline assessment (1) or not (0).

*Depressive symptoms* were measured at each assessment using the Edinburgh Postnatal Depression Scale (EPDS) [31], a measure often administered in South Africa [24,33,34]. The EPDS is a 10-item scale with 4 Likert-type responses for each item (maximum score of 30), with mothers’ self-reports indicating possible depressed mood with scores > = 13 (1) at every assessment (3, 6, 15, and 24 months) or not (0).

*Antenatal adherence* to 4 healthcare visits (1) or not (0) was assessed based on answers at the baseline and the 3-month assessment.

*Adherence to tasks to Prevent Mother-to-Child Transmission (PMTCT) by MLH*. HIV testing identified MLH during pregnancy, confirmed by self-report and by the government-issued Road to Health Card. All pregnant mothers were tested for HIV or were previously identified as HIV seropositive. We created a count of the following tasks the MLH completed: (a) exclusively breastfed for 6 months; (b) gave nevirapine at birth; (c) gave Bactrim for 6 weeks; (d) tested the child for HIV prior to 3 months of age; and (e) went to the clinic to receive the results of the baby’s HIV test. These were self-reported at each assessment and checked on the child’s Road to Health card to identify if each of the tasks were completed (1) or not (0).

*Adherence to ARV Medication for MLH*. At every time point, MLH were asked to rate their adherence to ARV medication on a scale ranging from poor to excellent. The outcome was the number of assessments (that is, 3, 6, 15, 24 months, range = 0 to 4) the mother reported “Very Good” or “Excellent” ARV adherence.

*Breastfeeding* for at least 6 months, which was self-reported by mothers and calculated as yes (0) or not (1) if breastfeeding at both the 3- and 6-month assessment or not (0).

*Having a low birthweight (LBW) infant* (that is, less than 2,500 grams = 0) or not (1) as recorded on the clinic or hospital birth registers or the child’s Road to Health Card.

*Having a stunted or malnourished child over 24 months (2 outcomes as occurring (1) or not (0) at any assessment at 3*, *6*, *15*, *or 24 months)*. Data collectors carried scales and weighed the child in kilograms and measured their length (centimeters) using a measuring mat. Infant anthropometric data were converted to z-scores based on the World Health Organization’s (WHO) age-adjusted norms for gender [35]. A Z-score below −2 was considered a serious growth deficit: <−2 for height-for-age z-scores (HAZ) was considered stunted (1) or never stunted (0). A standard deviation (SD) of <−2 for weight-for-age z-scores (WAZ) was considered underweight (1) or of weight >−2 SD was considered nourished (0).

*Securing the child support grant* by 6 months (0) or not (1).

*Immunizations* were classed as up to date if those expected were completed at the 6-, 15-, and 24-month time points, based on the South African government directives [36]. Adherence was documented on the child’s Road to Health card.

*Child hospitalizations* were recorded as occurring (1) if the child was ever hospitalized at the 3, 6-, 15-, and 24-month time points on the child’s Road to Health card or not (0).

*Developmental milestones* were based on the WHO stated developmental milestones [26,36] at 6, 15, and 24 months. The outcome was the count of developmental milestones the child completed at all the time points.

### Randomization

UCLA randomized matched clinic sites to the SC and the AC intervention conditions. There were 23 CHWs in the 4 SC clinics compared to 20 at the AC clinics; the number varied slightly due to resignations and hiring throughout the study. In particular, at one point in the study, a hiring freeze by the Provincial Dept. of Health resulted in a deficit of 6 CHWs over both conditions. At this point, the study agreed to pay the salaries of 6 new CHWs that the Dept. of Health hired, using their normal advertising, appointment, and training procedures. This was done in order to ensure that we were not evaluating the effect of a hiring freeze, but of CHWs’ implementation of home visiting in typical settings. These new CHWs received exactly the same 1-month training as the other CHWs who had been working from the start of the study.

### Intervention

The South African Dept. of Health employs CHWs as salaried, monthly workers with a minimum wage of R21.91 per hour. Each government CHWs was assigned to a specific clinic and the pregnant women living within the specific geographic area served by the clinic. Prior to the study, CHWs had previously received 10 days of in-service orientation and training by government supervisors when reassigned from clinic to community service. An additional month-long training was conducted by the Philani Mentor Mother Program prior to the clinic randomization, with trainers shadowing the CHWs during a 2-week period immediately afterwards on their early home visits to ensure good practice. CHWs provided consent to collect data on their sociodemographic characteristics.

All CHWs were trained to provide general information about the key perinatal health challenges: living with HIV, TB, reproductive health, danger signs during pregnancy, typical taught a process for engaging with the mothers—how to enter a house and bond with a mother, how to interview a mother, introduce oneself and one’s role, basic counseling skills, as well as how to monitor maternal and child status. This included growth monitoring, oral hydration, recognizing a child’s breathing problems, teaching the principles of good hygiene, and recording visits. After this training, the ongoing supervision and monitoring of home visits varied across conditions, as summarized in Table 1.

**Table 1:** Identification of responsibility for administrative and implementation functions of the CHWs and their supervisors.

	SC	AC
Hiring	Local government	Local government
Salary	Local government	Local government
Training	Initial Philani Model training	Initial Philani Model training
Visit documentation	Log sheet	CHWs log home visits on mobile phones and enter information about the pregnant women in a folder for supervisors’ monitoring
Supervision	Local government	Philani supervisors
Type of supervision	Log sheet	Random supervision visits every 2 weeks including real-time and data-informed feedback; support and reminders are sent to CHWs and supervisors by mobile phones.

AC, Accountable Care; CHWs, community health workers; SC, Standard Care.

#### SC condition

The CHWs were expected to be in the field conducting home visits for 4 days a week and to report to their clinic team leader 1 day a week. Paper records of the number of visits performed were to have been kept, but there were no supervisory visits in the field and no verification of whether visits took place or not. There was also no help with transport in medical emergencies. While we attempted to obtain records of visits and case assignments by CHWs, we were not successful.

#### AC condition

The CHWs implementation model in the AC was adapted from the Philani Maternal, Child Health, and Nutrition Programme [37], an evidence-based home visiting intervention model evaluated in 4 studies [24,33,38,39]. There were 3 differences between conditions: (1) CHWs in the AC received support materials to reinforce their prevention messages (backpacks with scales, thermometers, deworming medication, vitamin A, and condoms) and were required to document each home visit in paper logs and/or on mobile phones; (2) supervisors monitored daily implementation of both the paper/mobile reports and made home visits with the CHWs; and (3) supervisors had transport support for medical emergencies.

Two experienced Philani supervisors were allocated to provide supportive supervision to the CHWs allocated to the AC, even though the official supervisor remained the government clinic supervisor. Supportive supervision requires the following: the psychoeducational and interpersonal skills to both motivate and support CHWs to conduct effective home visits; the ability to identify and act effectively when either mothers or children are failing to maintain healthy routines; and the ability to hold CHWs accountable for not visiting mothers’ homes nor sharing key information about risk and protective factors for mothers and children. The supervisor randomly dropped in on the typical home visits on an ongoing basis (once every 2 weeks), covering 4 to 6 households a day. CHWs and supervisors then jointly identified at-risk children. These visits allowed them to support and validate the CHWs, as well as provide a role model for coping well with the mother on the home visit. CHWs in the AC group were expected to complete 6 home visits a day and were required to briefly log each visit onsite on a mobile phone with global positioning coordinates. Supervisors regularly checked the logging of home visits through an online portal and were able to quickly identify when CHWs were not out in the field, which was then addressed by a phone call or visit [37]. Supervisors had no power to fire or discipline CHWs; they reported personnel problems to government supervisors.

### Statistical analysis

Our primary research question was whether the intervention improved health outcomes. Because there were multiple outcomes of interest, we evaluated all 13 of them independently and created a primary outcome from those analyses. The primary outcome was the number of statistically significant intervention effects (defined as *p* < 0.025 using a one-sided test favoring the intervention) among the 13 outcomes; this strategy for assessing multiple outcomes in one test was developed by Harwood and colleagues [27]. Because these outcomes are likely correlated, we estimated a correlation of 0.10 as our average correlation between outcomes. Based on the Monte Carlo simulations run by Harwood and colleagues [27], 3 significant outcomes are needed to claim overall significance comparing the SC and the AC.

To evaluate the intervention’s effect on each of the 13 outcomes, we fit linear mixed effects model for the continuous outcomes and a logistic mixed effects model for each the binary outcomes. Because the intervention was assigned at the clinic level, we account for that with a random clinic effect; this is standard practice for cluster randomized trials. So, each mixed-effects model included the baseline clinic as a random effect and the intervention as a fixed effect. Because of computational difficulties and the prior assumption that there is clinic-to-clinic variation, we used a penalized likelihood approach for the clinic variance; this is equivalent to estimating via posterior mode with a weakly informative prior [40]. All models were fit with the blme package in R [40] using the default prior distributions on clinic variance. Using these models, we tested whether the outcome was improved in the intervention arm; if the *p*-value for that one-sided test is 0.025 or lower, it counts as a statistically significant result.

We estimated we had sufficient power of 0.80 to detect a small effect size of 0.21 overall omnibus test between the AC and SC conditions by 24 months. We assumed an intraclass area correlation of 0.01 and assumed 80% retention for the power analysis.

The study [28] was approved by the Stellenbosch Health Research Ethics Board (N16/05/064) by the UCLA Institutional Review Board (IRB#16–001362), and permission to recruit mothers to the study at primary care clinics was provided by the Eastern Cape Department of Health, South Africa. This study is reported as per the CONSORT extension for cluster trials.

## Results

Table 2 summarizes the baseline demographics of mothers in the AC versus SC conditions. Mothers were highly similar across conditions at baseline. Mothers were about 24 years old and had completed the ninth grade. Almost a quarter of women were married or were living with a partner; there were typically 3 adults and 3 children in a household. Most income was from government grants; only 16% of households reported income not derived solely from government grants. Food insecurity was common, with about 28% reporting moderate to severe food insecure. A total of 11 mothers (1.27%) reported making a suicide attempt at some point in their lifetime. The mean score for depressive symptoms (EPDS) is low, resulting in only about 4% of mothers having a depressed mood and 1% had a score indicating likely major depression. Chronic illness was experienced by about 1 in 10. Mothers typically had 3 lifetime sexual partners, with one in the last year. Alcohol prior to recognizing pregnancy was more common than after recognizing the pregnancy, a drop of about 2/3 of women drinking (4%). Only 2% reported problematic alcohol use, as reflected on the AUDIT-C. More than 80% of mothers had been tested for HIV in pregnancy, with 34% of the total sample being a MLH. More than half of the MLH were identified in this pregnancy (19%). Almost all were receiving ARV and more than 90% had disclosed to their partner. Fewer (about 75%) had disclosed to family members and it was rare to disclose to friends (6%). In their lifetime, 20% of women reported having experienced violence from a partner and only one-third less (15%) in the last year.

**Table 2:** Baseline characteristics of the sociodemographic characteristics and risk characteristics of the sample grouped by the Accountable Care (AC), Standard Care (SC) conditions, and Overall Total.

	AC	SC	Total
	(*n* = 450)	(*n* = 423)	(*n =* 873)
***Demographic Characteristics***			
Mothers mean Age in years (SD) (median, range)	25.3 (6.5) (24, 14–56)	25.6 (6.6) (24, 14–46)	25.5 (6.5) (24, 14–56)
Education in years, mean (SD)	9.13 (2.05)	9.59 (2.08)	9.35 (2.08)
Employment, n (%)	34 (8.1%)	18 (4.0%)	52 (6.0%)
Married or Lives with Partner n (%)	111 (24.89%)	100 (23.75%)	211 (24.34%)
Number of Adults in Household, Mean (SD)	3.23 (1.72)	3.38 (1.73)	3.3 (1.73)
Number of Children in Household, Mean (SD)	3.15 (2.01)	3.06 (2.1)	3.11 (2.05)
***Household Resources***			
EBIA Food Insecurity score, Mean (SD)	3.73 (3.7)	3.62 (3.74)	3.68 (3.72)
Food Insecurity Classifications n (%)			
Food Security	134 (30.0%)	136 (32.3%)	270 (31.14%)
Mild Food Insecurity	188 (42.15%)	164 (38.95%)	352 (40.6%)
Moderate Food Insecurity	81 (18.16%)	84 (19.95%)	165 (19.03%)
Severe Food Insecurity	43 (9.64%)	37 (8.79%)	80 (9.23%)
More Than 2 Days Hungry (Last 3 months) n (%)	33 (7.33%)	27 (6.38%)	60 (6.87%)
Any Household Receiving Non-Grant Income n (%)	46 (11.35%)	76 (21.35%)	122 (16.03%)
Receives Child Support Grant n (%)	393 (87.33%)	368 (87.00%)	761 (87.17%)
***Participant Health and Sexual Behavior***			
Ever Attempted Suicide n (%)	3 (0.67%)	8 (1.9%)	11 (1.27%)
EPDS Score, Mean (SD)	4.16 (4.12)	3.69 (4.08)	3.93 (4.11)
Depressed Mood (EPDS >13) Mean (SD)	14 (3.14)	18 (4.28)	32 (3.69)
Depression (EPDS > = 18) Mean (SD)	6 (1.35)	5 (1.19)	11 (1.27)
Chronic Illness n (%)	56 (12.56%)	44 (10.45%)	100 (11.53%)
Number of Times Pregnant, mean (SD)	1.29 (1.38)	1.2 (1.39)	1.25 (1.39)
Lifetime Number of Sexual Partners (Mean [**interquartile range IQR])	2.99 [2,4]	3.11 [2,4]	3.05 [2,4]
Last year Number of Sexual Partners (Mean [**interquartile range IQR])	1.11 [1,1]	1.13 [1,1]	1.12 [1,1]
***Alcohol/Tobacco Use***			
Use Alcohol Before Knowledge of Pregnancy n (%)	55 (12.33%)	59 (14.01%)	114 (13.15%)
Use Alcohol After Pregnancy Discovery n (%)	16 (3.59%)	19 (4.51%)	35 (4.04%)
AUDIT-C Score >2 Before Knowledge of Pregnancy n (%)	25 (5.61%)	25 (5.94%)	50 (5.77%)
AUDIT-C Score >2 After Knowledge of Pregnancy n (%)	9 (2.02%)	11 (2.61%)	20 (2.31%)
***HIV Status and Disclosure***			
Tested for HIV During Pregnancy n (%)	361 (80.94%)	345 (81.95%)	706 (81.43%)
vHIV Positive during pregnancy n (%)	74 (16.59%)	94 (22.33%)	168 (19.38%)
Mothers Living with HIV n (%)	142 (31.84%)	155 (36.82%)	297 (34.26%)
Ever Received ARVs n (%)	141 (99.3%)	153 (98.71%)	294 (98.99%)
Have Disclosed HIV Status n (%)	129 (90.21%)	147 (93.63%)	276 (92.00%)
Disclosed to Partner n (%)	88 (61.54%)	97 (61.78%)	185 (61.67%)
Disclosed to 1+ Family Members n (%)	106 74.13%)	118 (75.16%)	224 (74.67%)
Disclosed to 1+ Friends n (%)	11 (7.69%)	8 (5.10%)	19 (6.33%)
***Violence***			
Intimate Partner Violence–Lifetime n (%)	102 (22.87%)	89 (21.14%)	191 (22.03%)
Intimate Partner Violence–Recent n (%)	69 (15.47%)	61 (14.49%)	130 (14.99%

AC, Accountable Care; ARV, antiretroviral; AUDIT-C, Alcohol Use Disorders Identification Test-C; EBIA, Brazilian Household Food Insecurity Measurement Scale; EPDS, Edinburgh Postnatal Depression Scale; SC, Standard Care; SD, standard deviation.

### Implementation of home visits

There were no records to access for home visits in the SC. At 3 months, 7% (21/290) of mothers in the SC condition reported having been visited by a CHWs; 8% (24/312) reported visits at 6 months; 9% (27/283) at 15 months; and 8% (27/344) at 24 months.

Over the course of the study, CHWs in the AC condition had an average case load of 61 mother–infant pairs (range 27 to 110) from June 2017 to October 2021. This reflects CHWs visiting study participants, as well as additional women who became pregnant in their catchment area after the recruitment period for the study ended (between June 2017 and October 2018), but the post-birth follow-ups were continuing (until February 2021). The CHWs had an average caseload of 23 study participants, each (range 2 to 46), who were visited on average of 14 times, with 34% (1,735/5,104) supervised visits. In addition, at 3 months, 71% (244/314) of mothers in the AC condition reported CHWs home visits; 77% (271/354) at 6 months; 66% (232/353) at 15 months; and 62% (238/385) at 24 months.

### Outcomes

Table 3 summarizes the analysis of the main outcomes. We estimate AC improved the outcome for 11 of 13 outcomes; that is, the AC has a more positive impact on each outcome, except immunizations and adherence to antenatal visits. Confidence intervals reported are 95% two-sided intervals for the treatment effect (SC-AC); for continuous outcomes, they are on the same scale as the response; for binary outcomes, they are on the log-odds scale. We have a degree of certainty for the AC’s effect on ARV adherence (SC mean 2.3, AC mean 2.9, *p* < 0.025, 95% CI = [0.157, 1.578]), breastfeeding at 6 months (SC 39.7%, AC 49.4%, *p* = 0.032, 95% CI = [−0.022, 0.751]), reducing WAZ <−2 (SC 6.93%, AC 4.11%, *p* = 0.078, 95% CI = [−1.813, 0.291]), and developmental milestones (SC mean 18.5, AC mean 18.8, *p* = 0.068, 95% CI = [−0.084, 0.611]). Yet, because of the methodology identified prior to initiation of the study, we can only claim one significant outcome—ARV adherence. Thus, we fail to reach the critical value of 3 significant outcomes to identify a difference across the SC and AC conditions. We do not have sufficient evidence to reject the null hypothesis of no intervention effect.

**Table 3:** Summary of the measures contributing to the primary outcome, grouped by the SC condition and the AC condition^1^.

Outcome	Assessment Point	SC	AC	*p*-value	95% CI (AC-SC)
No Alcohol During Pregnancy (n/%)	Baseline	402/421 (95.5%)	430/446 (96.4%)	0.247	(−0.506, 1.048)
Ever Depressed (n/%)	All time points	23/130 (22.3%)	33/148 (22.3%)	0.431	(−0.707, 0.592)
>=4 Antenatal Visits (n/%)	3 Months	187/227 (82.4%)	196/253 (77.5%)	0.539	(−0.655, 0.602)
PMTCT Adherence Mean (SD)	3 and 6 Months	3.4 (0.8)	3.5 (0.8)	0.241	(−0.186, 0.393)
ARV Adherence Mean (SD)	3, 6, 15, 24 Months	2.3 (1.1)	2.9 (1.4)	0.008	(0.157, 1.576)
Breastfeeding (n/%)	6 Months	124/312 (39.7%)	176/356 (49.4%)	0.032	(−0.022, 0.751)
Low Birth Weight (n/%)	Baseline	39/280 (13.9%)	43/308 (14.0%)	0.397	(−0.669, 0.512)
Ever HAZ <−2 9 (n/%)	All Time Points	73/202 (36.1%)	82/219 (37.4%)	0.393	(−1.813, 0.291)
Ever WAZ <−2 (n/%)	All Time Points	14/202 (6.9%)	9/219 (4.1%)	0.078	(−0.596, 0.451)
Child Support Grant Secured (n/%)	6 Months	271/312 (86.9%)	309/356 (86.6%)	0.468	(−0509, 0.552)
Immunizations Up to Date (n/%)	6, 15, 24 Months	93/153 (60.8%)	94/159 (59.1%)	0.677	(−0.710, 0.441)
Ever Hospitalized (n/%)	3, 6, 15, 24 Months	140/359 (39.0%)	149/403 (37.0%)	0.299	(−0.478, 0.275)
Developmental Milestones (Mean/SD)	6, 15, 24 Months	18.5 (1.5)	18.8 (1.5)	0.068	(−0.084, 0.611)

^1^The reported *p*-values come from a one-sided test where the null hypothesis is no AC effect and the alternative is the AC improves the outcome. The 95% two-sided confidence interval is for the treatment effect (AC-SC); for binary outcomes, this is on the log-odds scale; for continuous outcomes, it is on the same scale as the response.

AC, Accountable Care; ARV, antiretroviral; HAZ, height-for-age z-scores; PMTCT, Prevention of Mother-to-Child Transmission of HIV; SC, Standard Care; SD, standard deviation; WAZ, weight-for-age z-scores.

## Discussion

In a real-world examination of the role of supportive supervision and monitoring, we did not find significant differences in maternal and child health outcomes between the AC versus the SC conditions. At 8 clinics in deeply rural South Africa, existing CHWs received enhanced training for a month and then were randomized by clinic to receive ongoing monitoring and supervision over time or standard supervision. Statistically, there were no benefits observed.

These findings contrast with earlier effectiveness data from 4 studies using a similar model of supervision and monitoring that found a range of significant benefits [24,33,37–39,41,42]. The Philani model, for example, was shown to have large improvements in the PMTCT tasks, longer breastfeeding, less malnutrition, decreases in maternal alcohol use and problematic alcohol use at 5 years post-birth and during pregnancy, lower maternal depression at 3 years post-birth, better early child growth, especially when mothers were depressed, fewer low birth weight babies, and mothers who were more sensitive to their children’s needs at 3 years of age compared to households without CHWs home visits [27,38,41,43–46]. These benefits were not present in this study. There are several possible explanations to account for the null findings.

In the previous effectiveness studies, the Philani program controlled of all aspects of the intervention (recruitment, training, supervision). Philani has developed implementation strategies refined over decades of experience [37]. The major difference between the Philani model and the CHWs implementation in this study was CHWs recruitment strategy. Philani created a recruitment system where communities put forward names of potential CHWs for their area, who were then interviewed by a panel including organization representatives from all levels of the organization. Philani narrowed the initial list to those who are positive role models in their communities, mothers who have similar adversities but have raised healthy children [47]. A pool of potential CHWs is then selected, monitored repeatedly and unexpectedly over multiple probationary months, and certified. Final decisions about who is finally hired is made based on experiences during the training and over the first few months of conducting home visits. These policies are typical of many private enterprise organizations, but not necessarily the government and nonprofit sectors [48].

In this study, all CHWs had already been hired by the Eastern Cape Department of Health, and most had been in their role for many years preceding the study. Before the study started, CHWs had predominantly been based in clinics and were reassigned at some point from 2011 to 2015 [13,18] to conduct home visits as part of the government’s plans for Re-engineering Health Care. It took substantial prompting and oversight by the Philani team to get CHWs to conduct the home visits. This did not occur in the SC, and, when asked, few mothers reported having received any home visits at all. This study suggests that recruitment procedures and monitoring of performance may be basic to effective implementation of CHWs programs.

A second limitation to the implementation was the ability to hold CHWs accountable and to release CHWs not meeting expectations. Because CHWs’ employer was the Department of Health, the Philani supervisors did not have the same leverage to dismiss inactive CHWs. While Philani did deliver negative performance reviews for some CHWs, the power to hold employees accountable rested with the Dept. of Health. It is not clear that it is easy to implement such accountability systems within large Depts. of Health—even though such systems are basic to sound implementation [19,49]. Both in South Africa [50] and, in general, in low-income countries [49], strikes are common among CHWs. There were at least 116 reports of more than 70 unique strikes in 23 low-income countries in an 8-year period [49]. The lack of trust and structural challenges for governments and health agencies to hold CHWs accountable is a major barrier to effective performance by CHWs.

Surprisingly, mothers in the deeply rural areas of Zithulele had far fewer risk behaviours than was the case in studies conducted in the Philani urban settings [28]. One of the reasons there are few significant outcomes may be the low rates of depression and alcohol use and the relatively high existing breastfeeding rates, high rates of securing child grants, and adherence to PMTCT tasks. This was not the case in any of the 4 previous studies evaluating the Philani program [26,37–39,42,51]. The quality and coverage of HIV care in this subdistrict, but also in South Africa more broadly, has improved significantly in the last 10 years [19,47]. Prior to study implementation, we expected rates of risk that are about 4 times higher and protective factors that were significantly lower based on data predominantly from peri-urban Cape Town. In peri-urban settings, the rates of depression have been documented at between 25% and 35% and alcohol use among mothers at 25% during pregnancy [22,24,38,52]. The rates were far lower in this study. Concurrently, about half of mothers in the SC condition breastfed for 6 months, compared to 3% in Cape Town. The child grant was obtained earlier and by almost all mothers in the Eastern Cape—goals not achieved by about 40% of mothers at 24 months post-birth in Cape Town. While there are many more hardships in daily life in rural communities (for example, carrying water, fewer households having flush toilets, few employment opportunities), there may also be some benefits to living in a rural setting. The low observed rates of risk may have significantly reduced our ability to detect differences associated with CHWs home visiting.

Many of our outcomes were based on self-reports, a potential source of bias by not having independent confirmation by clinic records or rapid diagnostic tests. Alcohol use, for example, may be potentially stigmatizing. However, we had rates of 25% in other rural areas [52] and in urban areas [24]. We could have confirmed alcohol use in the last 24 hours at a reasonable cost (<2 USD), but a reliable measure over the last 3 months is about 100 USD per participant—an unreasonable field cost. It would have been desirable to confirm mothers’ reports with clinic records, but in many cases, these records were incomplete and unavailable. For example, antenatal visit cards, which are assigned in pregnancy, are collected by clinics and then not available post-birth.

As outlined in our clinical trials registration [28], we utilized an analytic method aimed at considering multiple outcomes concurrently. One of the major advances of the South African Dept. of Health initiative to Re-engineer Primary Health Care is to shift CHWs from focusing on a single outcome to adopting a more generalized intervention approach. To claim overall statistical significance, we needed 3 or more of the outcomes to have *p*-values below 0.025. While this methodology has benefits, it still has setbacks, notably in the lack of nuance between nonsignificant outcomes. For example, a difference of 10% in breastfeeding over 6 months (*p* = 0.032) is treated the same as a difference of <0.1% in depressive-like symptoms (*p* = 0.431) in the primary outcome: Both are not significant at the 0.025 level. This arbitrary cutoff discards valuable information when creating the primary outcome.

To consider some alternative methods, we ran post hoc Monte Carlo simulations to see how significance may have been determined in alternative methods. If all outcomes have uniform 0.100 correlation and there are no intervention effects, then the probability we see 11 or more *positive* effects (*p* < 0.500) is 0.046 while the probability we see 1 more or significant (*p* < 0.025) effects is 0.325. Observing the mothers/children in the AC condition having better outcomes on 11 of 13 measures, as we had in this study, is highly unlikely under the null hypothesis. Another alternative could be to sum the Z-scores themselves rather than sum indicators form the Z-scores like *p* < 0.500 or *p* < 0.025.

New, alternative methodologies that account for the differences in outcomes (like breastfeeding and depressive-like symptoms) by quantifying uncertainty may be more appropriate for multi-outcome studies like this. Such analytic approaches are going to be needed to evaluate CHWs interventions that target multiple outcomes over time.

This study was pragmatic—recognizing that it is rarely possible to recruit CHWs from scratch—and seeing whether supportive, in-the-field supervision could demonstrate benefits to maternal and child health outcomes when working with already-employed government CHWs. We were fortunate that the trial had high internal and external validity in its implementation—all clinics in the district agreed to participate, as did the CHWs at each clinic, and recruitment and retention were high among the mothers. We were not able to definitively show that providing good quality supervision had a positive impact on the anticipated maternal and child health outcomes. Nevertheless, the investment of 2 experienced supervisors and a car and driver for every 20 CHWs created a support structure that ensured that CHWs were performing regular household visits—in short, doing the work for which they were paid for. Without this support structure, it seems like CHWs in rural areas do not perform regular home visits and may, therefore, not to be worth the investment. In addition, the lack of risk in deeply rural areas was unexpected, making evaluations using multiple indicators of maternal and child outcomes difficult to assess. In the future, both analytic strategies and reconfirming levels and the types of risks experienced will be critical in order to evaluate the efficacy of CHWs.

## Supporting information

S1 CONSORT Checklist CONSORT Checklist. (DOCX)

## Abbreviations

AC: Accountable Care
ARV: antiretroviral
AUDIT-C: Alcohol Use Disorders Identification Test-C
CHWs: community health workers
EPDS: Edinburgh Postnatal Depression Scale
HAZ: height-for-age z-scores
IQR: interquartile range
LBW: low birthweight
LMICs: low- and middle-income countries
MLH: mothers living with HIV
PMTCT: Prevention of Mother-to-Child Transmission of HIV
SC: Standard Care
SD: standard deviation
WAZ: weight-for-age z-scores
WHO: World Health Organization
