# Supplementary material for: The effect of supervision on community health workers’ effectiveness with households in rural South Africa: A cluster randomized controlled trial
Source: PLoS Med. 2023 Mar 2;20(3):e1004170. doi: 10.1371/journal.pmed.1004170 (PMC9980736; doi:10.1371/journal.pmed.1004170)
Supplement: S1 CONSORT Checklist — (DOCX) [file pmed.1004170.s001.docx]

**Supporting information**

Repository for data and R scripts used to generate estimates reported in this study can be found here: https://github.com/ruchitab24/HARI

We compiled a database of point prevalence surveys for hospital-associated drug resistant infections. The fields of this database along with their explanations are described below.

**GeoPosMethod**: indicates how the XY coordinates of a study were determined and entered: either the coordinates were given in the study (given); the coordinates were determined from the place name of the study (place name); or the centroid of multiple study site locations were entered (manual). If the samples have been collected from a whole province and no specific city/region is given, then the coordinates of the centroid are use (manual). If there is just a region of a country given such as “central” or “north”, then a random city in this area is chosen.

**GeoUncert**: If the coordinates were reported as a centroid (manual), the uncertainty in km was reported by taking the mean of uncertainty in the X and Y directions.

**InfectionType:** This refers to the type of infection extracted isolates were associated with (i.e. bloodstream, urinary tract infection, surgical site infection etc). If the associated infection was not reported, we report NA.

**PopCNTY**: We report the average population for each country averaged over all available years from 2010-2019.

**HRNTYR**: We report the hospitalization rates for each country, averaged over all available years from 2010-2019. (See Table B for rates and sources).

**HNinf (**N_i,j_): Refers to the number of infections reported by a particular hospital(s) over the study period.

**StudyPeriod**: Refers to the duration of the study period in days.

**HRes (**$P_{r,i,k,j})$**:** Refers to the proportion of clinical isolates from hospital-associated infections that are resistant to the corresponding drug-bug combination, for *E. coli*, *Klebsiella* spp., *Staphylococcus* spp., *Acinetobacter* spp., and *Pseudomonas* spp. using the study’s definition of resistance.

**DOI:** The Digital Object Identifier of the study included.

**Author:** The first author of the study’s last name.

**PubDate:** The year the article was published.

**ISO3:** The three-letter country code of the country the study took place in.

**Ycoord:** The latitude of the study location reported in decimal degrees, where positive values are north of the equator, negative ones are south of equator. The decimal degree is specified to at least two decimal degrees.

**Xcoord**: The longitude of the study location reported in decimal degrees, where **positive** values are east of the zero meridian and negative ones west of the zero meridian.

**StartDate**: The start date of study, as specified in the article, reported in dd/mm/yyyy format. If the specific day is not reported, the 15^th^ is assumed. If only the year is reported, then we assume the start date to be January 1^st^ of that year. The start date of the study often refers to the start date of the lab work/field work.

**EndDate**: The end date of study as specified in the article. Refer to start date for more detailed information on how to report dates.

**Species**: Refers to the species isolates are taken from. For the purposes of this study, only isolates from humans are taken from and so all entries will be classified as “Human” in our database.

**Setting**: For the purposes of this study, data will only be reported from hospital-associated settings and so all entries will be classified with “Hospital” in our database.

**SampleType**: Type of samples. Clinical samples generally include blood, urine, fecal etc.

**Pathogens**: The pathogen for which resistance rates are reported, which can include *E. coli*, *Klebsiella* spp., *Staphylococcus* spp., *Acinetobacter* spp., and *Pseudomonas* spp.

**Drug**: The antibiotic class tested if such data was reported. If this was not entered, we report NA. The 13 major classes of antibiotics considered include TET (Tetracyclines), PEN (Penicillins), SUL (Sulfonamides), MAC (Macrolides), AMI (Aminoglycides), FLA (Quinolones), CEP (Cephalosporins), AMP (Amphenicols), GLY (Glycopeptides), POL (Polymixins), CAR (Carbapenem), Monobactams (MON), and OTH (Other).

**Compound**: Refers to the specific antibiotic tested coded with a three-letter abbreviation, if this information is reported. If not, NA is entered. Please refer to S1 Table for a full list of antibiotic compounds, their corresponding drug class and ATCCS code.

**Bed**: Refers to the bed capacity of the hospital(s) where the study took place. If the bed capacity was not reported in the study itself, we searched for the bed capacity on hospital websites or contacted the corresponding author of the study.

**Fig A. Schematic of literature review and data extraction.** Boxes contain the number of articles identified, the reasons for exclusion from the initial search results, the number of original articles screened, the number of articles excluded because they were taken from national databases, the number of articles excluded because the combination of pathogen and drug resistance was unavailable, and the final number of articles used in the mapping process. The flow chart diagram was adapted from the PRISMA guidelines for reporting systematic reviews^1^.

**
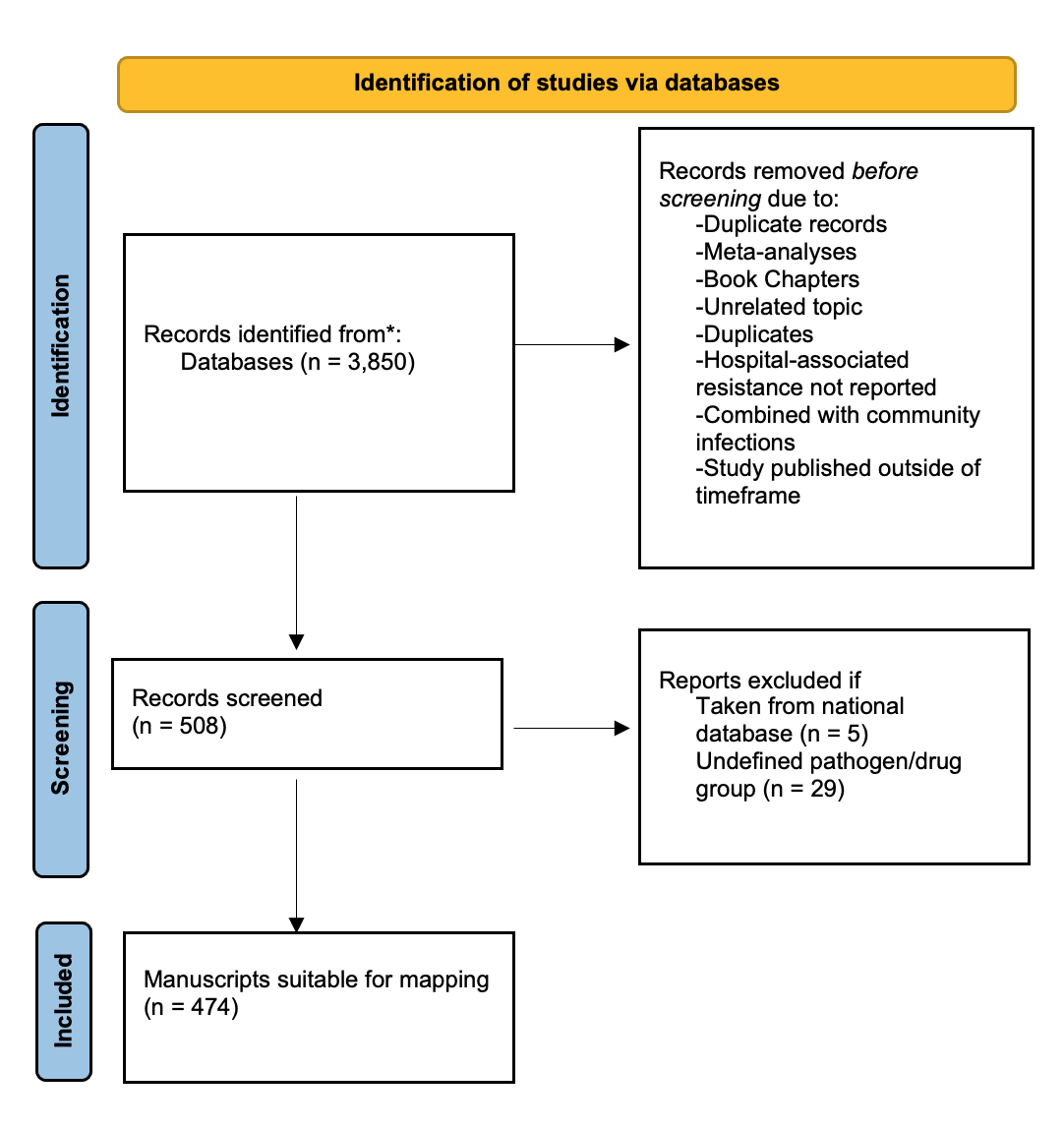
**

**Table A. Antibiotic designation and drug class.** For each antibiotic included in the analysis, a 3-letter code abbreviation of the drug, a 3-letter code abbreviation of the drug class, the full name of the drug, and the ATC Code for the drug were included.

| **Code** | **3-Letter code** | **Antibiotic Designation** | **ATC_Code** |
| --- | --- | --- | --- |
|  |  |  |  |
| **PEN** | AMC | Amoxicillin-Clavulanic acid | J01CR02 |
|  |  |  |  |
| **PEN** | AMP | Ampicillin | J01CA01 |
|  |  |  |  |
| **PEN** | AMX | Amoxicillin | J01CA04 |
|  |  |  |  |
| **PEN** | TIC | Ticarcillin | J01CA13 |
|  |  |  |  |
| **PEN** | CLO | Cloxacillin | J01CF02 |
|  |  |  |  |
| **PEN** | OXA | Oxacillin | J01CF04 |
|  |  |  |  |
| **PEN** | TIM | Ticarcillin-Clavulanic acid | J01CR03 |
|  |  |  |  |
| **PEN** | PIT | Piperacillin-Tazobactam | J01CR05 |
|  |  |  |  |
| **PEN** | SAM | Ampicillin-Sulbactam | J01CR01 |
|  |  |  |  |
| **PEN** | PES | Penicillin & Streptomycin | J01RA01 |
|  |  |  |  |
| **PEN** | MEC | Mecillinam | J01CA11 |
|  |  |  |  |
| **PEN** | PIP | Piperacillin | J01CA12 |
|  |  |  |  |
| **PEN** | FLU | Flucloxacillin | J01CF05 |
|  |  |  |  |
| **PEN** | CAR | Carbenicillin | J01CA03 |
|  |  |  |  |
| **PEN** | MET | Methicillin | J01CF03 |
|  |  |  |  |
| **PEN** | PEN | Penicillin | J01CE01 |
|  |  |  |  |
| **PEN** | CLA | Clavulanic acid | J01CR |
|  |  |  |  |
| **PEN** | TEM | Temocillin | J01CA17 |
|  |  |  |  |
| **PEN** | DIC | Dicloxacillin | QJ51CF01 |
|  |  |  |  |
| **PEN** | NAF | Nafcillin | J01CF06 |
|  |  |  |  |
| **CEP** | CRO | Ceftriaxone | J01DD04 |
|  |  |  |  |
| **CEP** | CAZ | Ceftazidime | J01DD02 |
|  |  |  |  |
| **CEP** | CLX | Cefalexin | J01DB01 |
|  |  |  |  |
| **CEP** | CTX | Cefotaxime | J01DD01 |
|  |  |  |  |
| **CEP** | FEP | Cefepime | J01DE01 |
|  |  |  |  |
| **CEP** | FOX | Cefoxitin | J01DC01 |
|  |  |  |  |
| **CEP** | CFL | Cefalotin | J01DB03 |
|  |  |  |  |
| **CEP** | CFU | Ceftiofur | QJ01DD90 |
|  |  |  |  |
| **CEP** | CXM | Cefuroxime | J01DC02 |
|  |  |  |  |
| **CEP** | CPD | Cefpodoxime | J01DD13 |
|  |  |  |  |
| **CEP** | CFZ | Cefazolin | J01DB04 |
|  |  |  |  |
| **CEP** | CFM | Cefixime | J01DD08 |
|  |  |  |  |
| **CEP** | CMD | Cefamandole | J01DC03 |
|  |  |  |  |
| **CEP** | CZX | CAZ/CTX | J01DD51 |
|  |  |  |  |
| **CEP** | CFP | Cefoperazone | J01DD12 |
|  |  |  |  |
| **CEP** | MOX | Moxalactam | J01DD06 |
|  |  |  |  |
| **CEP** | CPO | Cefpirome | J01DE02 |
|  |  |  |  |
| **CEP** | CTT | Cefotetan | J01DC05 |
|  |  |  |  |
| **CEP** | CFR | Cefradine | J01DB09 |
|  |  |  |  |
| **CEP** | CPT | Ceftaroline | J01DI02æ |
|  |  |  |  |
| **CEP** | CBP | Ceftobiprole | J01DI01 |
|  |  |  |  |
| **CEP** | CTT | Cefotetan | J01DC05 |
|  |  |  |  |
| **CEP** | CFQ | Cefquinome | QJ01DE90 |
|  |  |  |  |
| **CEP** | CPR | Cephradine | J01DB09 |
|  |  |  |  |
| **CEP** | SFP | Sulbactam+Cefoperazone | NA |
|  |  |  |  |
| **CEP** | CZM | Ceftizoxime | J01DD07 |
|  |  |  |  |
| **CEP** | CLD | Cephaloridine | J01DB02 |
|  |  |  |  |
| **CEP** | CLM | Cefalonium | QJ51DB90 |
|  |  |  |  |
| **MON** | ATM | Aztreonam | J01DF01 |
|  |  |  |  |
| **CAR** | IPM | Imipenem | J01DH51 |
|  |  |  |  |
| **CAR** | ERT | Ertapenem | J01DH03 |
|  |  |  |  |
| **CAR** | MEM | Meropenem | J01DH02 |
|  |  |  |  |
| **CAR** | DOR | Doripenem | J01DH04 |
|  |  |  |  |
| **AMI** | KAN | Kanamycin | J01GB04 |
|  |  |  |  |
| **AMI** | GEN | Gentamicin | J01GB03 |
|  |  |  |  |
| **AMI** | NEO | Neomycin | J01GB05 |
|  |  |  |  |
| **AMI** | STR | Streptomycin | J01GA01 |
|  |  |  |  |
| **AMI** | AMK | Amikacin | J01GB06 |
|  |  |  |  |
| **AMI** | TOB | Tobramycin | J01GB01 |
|  |  |  |  |
| **AMI** | APR | Apramycin | QA07AA92 |
|  |  |  |  |
| **AMI** | NET | Netilmicin | J01GB07 |
|  |  |  |  |
| **AMI** | SPT | Spectinomycin | J01XX04 |
|  |  |  |  |
| **AMI** | ISP | Isepamicin | J01GB11æ |
|  |  |  |  |
| **QUI** | CIP | Ciprofloxacin | J01MA02 |
|  |  |  |  |
| **QUI** | NAL | Nalidixic acid | J01MB02 |
|  |  |  |  |
| **QUI** | ENR | Enrofloxacin | QJ01MA90 |
|  |  |  |  |
| **QUI** | NOR | Norfloxacin | J01MA06 |
|  |  |  |  |
| **QUI** | OFX | Ofloxacin | J01MA01 |
|  |  |  |  |
| **QUI** | OXO | Oxolinic acid | J01MB05 |
|  |  |  |  |
| **QUI** | FLQ | Flumequine | J01MB07 |
|  |  |  |  |
| **QUI** | MXF | Moxifloxacin | J01MA14 |
|  |  |  |  |
| **QUI** | LVX | Levofloxacin | J01MA12 |
|  |  |  |  |
| **QUI** | PEF | Pefloxacin | J01MA03 |
|  |  |  |  |
| **QUI** | OLA | Olaquindox | NA |
|  |  |  |  |
| **QUI** | MEQ | Mequindox | NA |
|  |  |  |  |
| **QUI** | MRB | Marbofloxacin | QJ01MA93 |
|  |  |  |  |
| **QUI** | GAT | Gatifloxacin | S01AE0E |
|  |  |  |  |
| **QUI** | LOM | Lomefloxacin | J01MA07 |
|  |  |  |  |
| **QUI** | DAN | Danofloxacin | QJ01MA92 |
|  |  |  |  |
| **AMP** | CHL | Chloramphenicol | J01BA01 |
|  |  |  |  |
| **AMP** | FFC | Florfenicol | QJ01BA90 |
|  |  |  |  |
| **AMP** | TFC | Tiafenicol | J01BA02 |
|  |  |  |  |
| **TET** | TET | Tetracycline | J01AA07 |
|  |  |  |  |
| **TET** | OXT | Oxytetracycline | J01AA06 |
|  |  |  |  |
| **TET** | DOX | Doxycycline | J01AA02 |
|  |  |  |  |
| **TET** | MIN | Minocycline | J01AA08 |
|  |  |  |  |
| **TET** | TIG | Tigecycline | J01AA12 |
|  |  |  |  |
| **TET** | DOG | Doxycycline & Gentamicin | NA |
|  |  |  |  |
| **TET** | CTE | Chlortetracycline | J01AA03 |
|  |  |  |  |
| **SUL** | SXT | Sulfamethoxazole-Trimethoprim | J01EE01 |
|  |  |  |  |
| **SUL** | SMZ | Sulfamethoxazole | J01EC01 |
|  |  |  |  |
| **SUL** | SOX | Sulfafurazole/Sulfisoxazole | J01EB05 |
|  |  |  |  |
| **SUL** | SUT | Sulfonamides-Trimethoprim | J01EE |
|  |  |  |  |
| **SUL** | SSS | Sulphonamides | J01E |
|  |  |  |  |
| **SUL** | TDZ | Trimethoprim+sulfadiazine | QJ01EW10 |
|  |  |  |  |
| **SUL** | TMP | Trimethoprim | J01EA01 |
|  |  |  |  |
| **SUL** | SMN | Sulfamonomethoxine | QJ01EQ18 |
|  |  |  |  |
| **MAC** | ERY | Erythromycin | J01FA01 |
|  |  |  |  |
| **MAC** | LIN | Lincomycin | J01FF02 |
|  |  |  |  |
| **MAC** | CLI | Clindamycin | J01FF01 |
|  |  |  |  |
| **MAC** | CLR | Clarithromycin | J01FA09 |
|  |  |  |  |
| **MAC** | TYL | Tylosin | QJ01FA90 |
|  |  |  |  |
| **MAC** | AZM | Azithromycin | J01FA10 |
|  |  |  |  |
| **MAC** | SPI | Spiramycin | J01FA02 |
|  |  |  |  |
| **MAC** | TIL | Tilmicosin | QJ01FA91 |
|  |  |  |  |
| **MAC** | ROX | Roxithromycin | J01FA06 |
|  |  |  |  |
| **GLY** | VAN | Vancomycin | J01XA01 |
|  |  |  |  |
| **GLY** | TEC | Teicoplanin | J01XA02 |
|  |  |  |  |
| **GLY** | AVO | Avoparicin | NA |
|  |  |  |  |
| **POL** | PMB | Polymixin (B) | J01XB02 |
|  |  |  |  |
| **POL** | CST | Colistin | J01XB01 |
|  |  |  |  |
| **OTH** | LIZ | Linezolid | J01XX08 |
|  |  |  |  |
| **OTH** | NIT | Nitrofurantoin | J01XE01 |
|  |  |  |  |
| **OTH** | RIF | Rifampicin | J04AB02 |
|  |  |  |  |
| **OTH** | Q-D | Quinupristin-Dalfopristin | J01FG02 |
|  |  |  |  |
| **OTH** | BAC | Bacitracin | J01XX10 |
|  |  |  |  |
| **OTH** | FUR | Furazidin | J01XE03 |
|  |  |  |  |
| **OTH** | DAP | Daptomycin | J01XX09 |
|  |  |  |  |
| **OTH** | MUP | Mupirocin | D06AX09 |
|  |  |  |  |
| **OTH** | FOF | Fosfomycin | J01XX01 |
|  |  |  |  |
| **OTH** | FUS | Fusidic acid | J01XC01 |
|  |  |  |  |
| **OTH** | MTD | Metronidazole | J01XD01 |
|  |  |  |  |
| **OTH** | PRI | Pristinamycin | J01FG01 |
|  |  |  |  |
| **OTH** | CRB | Carbadox | NA |
|  |  |  |  |
| **OTH** | FRZ | Furazolidone | QJ01XE90 |
|  |  |  |  |
| **OTH** | TIA | Tiamulin | QJ01XQ01 |
|  |  |  |  |
| **OTH** | NOV | Novobiocin | QJ01XX95 |
|  |  |  |  |

**Table B. Hospitalization rates per country and sources.** Hospitalization rates refer to the number of hospitalizations that occur per population per year. For each country, the 3-letter country code, full country name, hospitalization rate (per person-year), the number of years for which hospitalization rates were available (where hospitalization rates were collected between 2010 and 2019), and the source of this data are included.

| **Country ISO3** | **Country** | **Hospitalization Rates** | **Years** | **Source** |
| --- | --- | --- | --- | --- |
| **ALB** | Albania | 0.090 | 2010-2019 | 2 |
| **ARG** | Argentina | 0.055 | 2015 | 3 |
| **AUS** | Australia | 0.165 | 2010-2019 | 4-6 |
| **AUT** | Austria | 0.262 | 2010-2018 | 7 |
| **BEL** | Belgium | 0.169 | 2010-2014, 2016-2018 | 7 |
| **BGD** | Bangladesh | 0.024 | 2011 | 8 |
| **BHR** | Bahrain | 0.083 | 2010-2017 | 9,10 |
| **BIH** | Bosnia and Herzegovina | 0.113 | 2010-2016 | 11 |
| **BOL** | Bolivia | 0.066 | 2010-2019 | 12 |
| **BRA** | Brazil | 0.055 | 2012 | 3 |
| **BTN** | Bhutan | 0.086 | 2010-2019 | 13 |
| **CAN** | Canada | 0.078 | 2010-2019 | 14 |
| **CHE** | Switzerland | 0.170 | 2010-2019 | 15 |
| **CHL** | Chile | 0.093 | 2010-2018 | 7 |
| **CHN** | China | 0.156 | 2011-2019 | 16,17 |
| **COD** | Dem Rep of Congo | 0.034 | 2010-2011 | 18 |
| **COL** | Colombia | 0.064 | 2017 | 19 |
| **CPV** | Cabo Verde | 0.051 | 2010-2018 | 20 |
| **CRI** | Costa Rica | 0.056 | 2010-2019 | 7 |
| **CYP** | Cyprus | 0.072 | 2010-2011, 2013-2018 | 21 |
| **CZE** | Czechia | 0.202 | 2010-2018 | 7 |
| **DZA** | Algeria | 0.077 | 2010-2012 | 22 |
| **DEU** | Germany | 0.252 | 2010-2018 | 7 |
| **ECU** | Ecuador | 0.072 | 2010-2019 | 23 |
| **EGY** | Egypt | 0.068 | 2000 | 24 |
| **ESP** | Spain | 0.104 | 2010-2019 | 7 |
| **ETH** | Ethiopia | 0.010 | 2012 | 25 |
| **FIN** | Finland | 0.173 | 2010-2018 | 7 |
| **FRA** | France | 0.173 | 2010-2019 | 7 |
| **GAB** | Gabon | 0.050 | 2012 | 26 |
| **GBR** | United Kingdom | 0.129 | 2010-2016, 2018 | 7 |
| **GEO** | Georgia | 0.115 | 2010-2019 | 27 |
| **GRC** | Greece | 0.175 | 2010-2014 | 7 |
| **HRV** | Croatia | 0.175 | 2010-2018 | 21 |
| **IDN** | Indonesia | 0.033 | 2010-2019 | 28 |
| **IND** | India | 0.033 | 2014, 2017 | 29,30 |
| **IRL** | Ireland | 0.135 | 2010-2019 | 7 |
| **IRN** | Iran | 0.064 | 2012-2019 | 31 |
| **IRQ** | Iraq | 0.085 | 2010, 2015, 2019 | 32 |
| **ISR** | Israel | 0.160 | 2010-2018 | 7 |
| **ITA** | Italy | 0.123 | 2010-2018 | 7 |
| **JAM** | Jamaica | 0.017 | 2015 | 33 |
| **JOR** | Jordan | 0.042 | 2010-2019 | 34,35 |
| **JPN** | Japan | 0.122 | 2011, 2014, 2017 | 7 |
| **KAZ** | Kazakhstan | 0.151 | 2010-2019 | 27 |
| **KEN** | Kenya | 0.038 | 2013 | 36 |
| **KHM** | Cambodia | 0.047 | 2010-2017, 2019 | 37 |
| **KOR** | South Korea | 0.161 | 2010-2018 | 7 |
| **LBY** | Libya | 0.078 | 2013-2014 | 38 |
| **LKA** | Sri Lanka | 0.274 | 2011 | 8 |
| **MAR** | Morocco | 0.035 | 2010-2017 | 39 |
| **MEX** | Mexico | 0.048 | 2010-2018 | 7 |
| **MMR** | Myanmar | 0.043 | 2010-2017 | 40 |
| **MNE** | Montenegro | 0.120 | 2019 | 21 |
| **MNG** | Mongolia | 0.265 | 2010-2019 | 41 |
| **MYS** | Malaysia | 0.114 | 2010-2018 | 42 |
| **NLD** | Netherlands | 0.112 | 2010-2019 | 7 |
| **NOR** | Norway | 0.164 | 2010-2019 | 7 |
| **NPL** | Nepal | 0.013 | 2010 | 43 |
| **PAN** | Panama | 0.039 | 2010-2018 | 44 |
| **PER** | Peru | 0.029 | 2010-2016 | 45 |
| **POL** | Poland | 0.170 | 2010-2019 | 7 |
| **PRT** | Portugal | 0.085 | 2010-2015, 2018 | 7 |
| **PRY** | Paraguay | 0.037 | 2010-2018 | 46 |
| **ROU** | Romania | 0.230 | 2010-2013 | 27 |
| **RUS** | Russia | 0.213 | 2010-2019 | 27 |
| **SGP** | Singapore | 0.088 | 2011 | 8 |
| **SRB** | Serbia | 0.154 | 2010-2014 | 27 |
| **SVK** | Slovakia | 0.193 | 2010-2012, 2014 | 27 |
| **SVN** | Slovenia | 0.178 | 2010-2019 | 7 |
| **THA** | Thailand | 0.046 | 2010, 2012, 2014, 2016, 2018 | 47 |
| **TUR** | Turkey | 0.161 | 2010-2018 | 7 |
| **TWN** | Taiwan | 0.124 | 2010-2019 | 48 |
| **UGA** | Uganda | 0.045 | 2015-2019 | 49 |
| **UKR** | Ukraine | 0.214 | 2010-2015 | 27 |
| **USA** | United States | 0.114 | 2010-2019 | 50 |
| **VNM** | Vietnam | 0.120 | 2015 | 8 |
| **ZAF** | South Africa | 0.053 | 2013 | 51 |
| **ZMB** | Zambia | 0.030 | 2013 | 52 |
| **ZWE** | Zimbabwe | 0.030 | 2015 | 53 |

**Table C. Number of HARIs per year by country.** For each country, the 3-letter country code, full country name, the number of HARIs per year, and the lower and upper 95% Credible Intervals are included.

| **Country IS03** | **Country** | **HARIs per year** | **95% CrI Lower** | **95% CrI Upper** |
| --- | --- | --- | --- | --- |
| **ABW** | Aruba | 1200 | 333 | 2060 |
| **AFG** | Afghanistan | 152000 | 0 | 354000 |
| **AGO** | Angola | 549000 | 95500 | 1000000 |
| **AIA** | Anguilla | 0 | 0 | 0 |
| **ALB** | Albania | 73100 | 12700 | 133000 |
| **ALD** | Åland Islands | 0 | 0 | 0 |
| **AND** | Andorra | 0 | 0 | 0 |
| **ARE** | United Arab Emirates (the) | 110000 | 30600 | 189000 |
| **ARG** | Argentina | 758000 | 149000 | 1370000 |
| **ARM** | Armenia | 51100 | 8880 | 93300 |
| **ASM** | American Samoa | 0 | 0 | 0 |
| **ATC** | Antarctica | 0 | 0 | 0 |
| **ATF** | French Southern Territories (the) | 0 | 0 | 0 |
| **ATG** | Antigua and Barbuda | 1090 | 304 | 1880 |
| **AUS** | Australia | 413000 | 115000 | 711000 |
| **AUT** | Austria | 99800 | 27800 | 172000 |
| **AZE** | Azerbaijan | 173000 | 30100 | 316000 |
| **BDI** | Burundi | 46000 | 0 | 107000 |
| **BEL** | Belgium | 146000 | 40300 | 251000 |
| **BEN** | Benin | 365000 | 0 | 758000 |
| **BFA** | Burkina Faso | 81100 | 0 | 189000 |
| **BGD** | Bangladesh | 304000 | 99100 | 508000 |
| **BGR** | Bulgaria | 120000 | 20900 | 220000 |
| **BHR** | Bahrain | 13400 | 3740 | 23100 |
| **BHS** | Bahamas (the) | 4380 | 1220 | 7540 |
| **BIH** | Bosnia and Herzegovina | 111000 | 19400 | 203000 |
| **BLM** | Saint Barthélemy | 0 | 0 | 0 |
| **BLR** | Belarus | 163000 | 28400 | 298000 |
| **BLZ** | Belize | 6740 | 1170 | 12300 |
| **BMU** | Bermuda | 719 | 200 | 1240 |
| **BOL** | Bolivia | 227000 | 39400 | 414000 |
| **BRA** | Brazil | 3470000 | 602000 | 6330000 |
| **BRB** | Barbados | 3230 | 899 | 5560 |
| **BRN** | Brunei Darussalam | 4870 | 1360 | 8390 |
| **BTN** | Bhutan | 19600 | 3410 | 35800 |
| **BWA** | Botswana | 39800 | 6920 | 72600 |
| **CAF** | Central African Republic (the) | 18900 | 0 | 44100 |
| **CAN** | Canada | 289000 | 80600 | 498000 |
| **CHE** | Switzerland | 144000 | 40100 | 247000 |
| **CHL** | Chile | 174000 | 48400 | 299000 |
| **CHN** | China | 52300000 | 10100000 | 94500000 |
| **CIV** | Côte d'Ivoire | 444000 | 77200 | 811000 |
| **CMR** | Cameroon | 298000 | 95100 | 501000 |
| **COD** | Congo (the Democratic Republic of the) | 89100 | 22400 | 156000 |
| **COG** | Congo (the) | 92900 | 16200 | 170000 |
| **COK** | Cook Islands (the) | 0 | 0 | 0 |
| **COL** | Colombia | 962000 | 167000 | 1760000 |
| **COM** | Comoros (the) | 14700 | 2550 | 26800 |
| **CPV** | Cabo Verde | 8370 | 1460 | 15300 |
| **CRI** | Costa Rica | 84400 | 14700 | 154000 |
| **CUB** | Cuba | 0 | 0 | 0 |
| **CUW** | Curaçao | 1770 | 494 | 3050 |
| **CYM** | Cayman Islands (the) | 730 | 204 | 1260 |
| **CYP** | Cyprus | 8510 | 2370 | 14700 |
| **CZE** | Czechia | 213000 | 59200 | 366000 |
| **DEU** | Germany | 2060000 | 575000 | 3550000 |
| **DJI** | Djibouti | 16800 | 2920 | 30700 |
| **DMA** | Dominica | 1240 | 216 | 2260 |
| **DNK** | Denmark | 65400 | 18200 | 113000 |
| **DOM** | Dominican Republic (the) | 185000 | 32200 | 339000 |
| **DZA** | Algeria | 990000 | 172000 | 1810000 |
| **ECU** | Ecuador | 373000 | 64900 | 682000 |
| **EGY** | Egypt | 2040000 | 354000 | 3720000 |
| **ERI** | Eritrea | 0 | 0 | 0 |
| **ESB** | Western Sahara | 0 | 0 | 0 |
| **ESP** | Spain | 464000 | 129000 | 799000 |
| **EST** | Estonia | 14900 | 4160 | 25700 |
| **ETH** | Ethiopia | 69200 | 17400 | 121000 |
| **FIN** | Finland | 94300 | 26300 | 162000 |
| **FJI** | Fiji | 15400 | 2670 | 28100 |
| **FLK** | Falkland Islands (the) [Malvinas] | 0 | 0 | 0 |
| **FRA** | France | 1120000 | 313000 | 1940000 |
| **FRO** | Faroe Islands (the) | 0 | 0 | 0 |
| **FSM** | Micronesia (Federated States of) | 1970 | 342 | 3590 |
| **GAB** | Gabon | 32400 | 5640 | 59200 |
| **GBR** | United Kingdom | 882000 | 246000 | 1520000 |
| **GEO** | Georgia | 138000 | 23900 | 251000 |
| **GGY** | Guernsey | 0 | 0 | 0 |
| **GHA** | Ghana | 525000 | 91300 | 959000 |
| **GIB** | Gibraltar | 0 | 0 | 0 |
| **GIN** | Guinea | 51000 | 0 | 119000 |
| **GMB** | Gambia (the) | 9370 | 0 | 21800 |
| **GNB** | Guinea-Bissau | 7670 | 0 | 17900 |
| **GNQ** | Equatorial Guinea | 13800 | 2810 | 24900 |
| **GRC** | Greece | 190000 | 53000 | 328000 |
| **GRD** | Grenada | 1930 | 336 | 3530 |
| **GRL** | Greenland | 0 | 0 | 0 |
| **GTM** | Guatemala | 287000 | 49800 | 523000 |
| **GUM** | Guam | 0 | 0 | 0 |
| **GUY** | Guyana | 13500 | 2350 | 24700 |
| **HKG** | Hong Kong | 84400 | 23500 | 145000 |
| **HMD** | Heard Island and McDonald Islands | 0 | 0 | 0 |
| **HND** | Honduras | 168000 | 29300 | 307000 |
| **HRV** | Croatia | 70200 | 19600 | 121000 |
| **HTI** | Haiti | 194000 | 33800 | 355000 |
| **HUN** | Hungary | 110000 | 30600 | 189000 |
| **IDN** | Indonesia | 946000 | 277000 | 1610000 |
| **IMN** | Isle of Man | 951 | 265 | 1640 |
| **IND** | India | 8910000 | 2580000 | 15200000 |
| **IOT** | British Indian Ocean Territory (the) | 0 | 0 | 0 |
| **IRL** | Ireland | 65100 | 18100 | 112000 |
| **IRN** | Iran | 1580000 | 275000 | 2880000 |
| **IRQ** | Iraq | 223000 | 69900 | 377000 |
| **ISL** | Iceland | 4060 | 1130 | 6990 |
| **ISR** | Israel | 143000 | 39800 | 246000 |
| **ITA** | Italy | 732000 | 204000 | 1260000 |
| **JAM** | Jamaica | 15000 | 2600 | 27300 |
| **JEY** | Jersey | 0 | 0 | 0 |
| **JOR** | Jordan | 129000 | 23900 | 234000 |
| **JPN** | Japan | 994000 | 277000 | 1710000 |
| **KAZ** | Kazakhstan | 837000 | 146000 | 1530000 |
| **KEN** | Kenya | 586000 | 162000 | 1010000 |
| **KGZ** | Kyrgyzstan | 111000 | 19400 | 204000 |
| **KHM** | Cambodia | 231000 | 40200 | 422000 |
| **KIR** | Kiribati | 2030 | 353 | 3710 |
| **KNA** | Saint Kitts and Nevis | 594 | 166 | 1020 |
| **KOR** | South Korea | 821000 | 229000 | 1410000 |
| **KWT** | Kuwait | 47300 | 13200 | 81400 |
| **LAO** | Lao People's Democratic Republic (the) | 124000 | 21500 | 226000 |
| **LBN** | Lebanon | 118000 | 20600 | 216000 |
| **LBR** | Liberia | 19700 | 0 | 45900 |
| **LBY** | Libya | 158000 | 27400 | 288000 |
| **LCA** | Saint Lucia | 3160 | 549 | 5760 |
| **LIE** | Liechtenstein | 0 | 0 | 0 |
| **LKA** | Sri Lanka | 1780000 | 310000 | 3260000 |
| **LSO** | Lesotho | 36700 | 6380 | 67000 |
| **LTU** | Lithuania | 31300 | 8730 | 53900 |
| **LUX** | Luxembourg | 6970 | 1940 | 12000 |
| **LVA** | Latvia | 21500 | 5990 | 37000 |
| **MAC** | Macao | 7200 | 2010 | 12400 |
| **MAF** | Saint Martin (French part) | 0 | 0 | 0 |
| **MAR** | Morocco | 459000 | 0 | 965000 |
| **MCO** | Monaco | 0 | 0 | 0 |
| **MDA** | Moldova (the Republic of) | 45900 | 7980 | 83800 |
| **MDG** | Madagascar | 495000 | 0 | 1700000 |
| **MDV** | Maldives | 9170 | 1590 | 16700 |
| **MEX** | Mexico | 1410000 | 337000 | 2480000 |
| **MHL** | Marshall Islands (the) | 1020 | 176 | 1850 |
| **MKD** | Republic of North Macedonia | 36000 | 6250 | 65700 |
| **MLI** | Mali | 78500 | 0 | 183000 |
| **MLT** | Malta | 5650 | 1570 | 9730 |
| **MMR** | Myanmar | 618000 | 101000 | 1140000 |
| **MNE** | Montenegro | 22300 | 3870 | 40700 |
| **MNG** | Mongolia | 255000 | 44400 | 466000 |
| **MNP** | Northern Mariana Islands (the) | 0 | 0 | 0 |
| **MOZ** | Mozambique | 121000 | 0 | 282000 |
| **MRT** | Mauritania | 78100 | 13600 | 143000 |
| **MSR** | Montserrat | 0 | 0 | 0 |
| **MUS** | Mauritius | 14200 | 3970 | 24500 |
| **MWI** | Malawi | 74300 | 0 | 173000 |
| **MYS** | Malaysia | 764000 | 109000 | 1420000 |
| **NAM** | Namibia | 43100 | 7490 | 78600 |
| **NCL** | New Caledonia | 0 | 0 | 0 |
| **NER** | Niger (the) | 93000 | 0 | 217000 |
| **NFK** | Norfolk Island | 0 | 0 | 0 |
| **NGA** | Nigeria | 2500000 | 452000 | 4550000 |
| **NIC** | Nicaragua | 113000 | 19600 | 206000 |
| **NIU** | Niue | 0 | 0 | 0 |
| **NLD** | Netherlands | 191000 | 53400 | 330000 |
| **NOR** | Norway | 82300 | 22800 | 142000 |
| **NPL** | Nepal | 168000 | 47000 | 289000 |
| **NRU** | Nauru | 141 | 39.4 | 244 |
| **NZL** | New Zealand | 55300 | 15400 | 95200 |
| **OMN** | Oman | 55900 | 15600 | 96300 |
| **PAK** | Pakistan | 10000000 | 1910000 | 18100000 |
| **PAN** | Panama | 16300 | 4550 | 28100 |
| **PCN** | Pitcairn | 0 | 0 | 0 |
| **PER** | Peru | 281000 | 48900 | 514000 |
| **PHL** | Philippines (the) | 1870000 | 325000 | 3410000 |
| **PLW** | Palau | 202 | 56.4 | 349 |
| **PNG** | Papua New Guinea | 152000 | 26300 | 277000 |
| **POL** | Poland | 562000 | 157000 | 967000 |
| **PRI** | Puerto Rico | 35900 | 10000 | 61800 |
| **PRK** | Korea (the Democratic People's Republic of) | 0 | 0 | 0 |
| **PRT** | Portugal | 86100 | 24000 | 148000 |
| **PRY** | Paraguay | 77800 | 13500 | 142000 |
| **PSX** | Palestine, State of | 0 | 0 | 0 |
| **PYF** | French Polynesia | 0 | 0 | 0 |
| **QAT** | Qatar | 31800 | 8870 | 54800 |
| **ROU** | Romania | 441000 | 123000 | 759000 |
| **RUS** | Russia | 7040000 | 942000 | 13100000 |
| **RWA** | Rwanda | 39800 | 10000 | 69700 |
| **SAU** | Saudi Arabia | 385000 | 107000 | 663000 |
| **SDN** | Sudan (the) | 171000 | 0 | 398000 |
| **SEN** | Senegal | 281000 | 48900 | 514000 |
| **SGP** | Singapore | 49500 | 13800 | 85200 |
| **SGS** | South Georgia and the South Sandwich Islands | 0 | 0 | 0 |
| **SHN** | Saint Helena, Ascension and Tristan da Cunha | 0 | 0 | 0 |
| **SLB** | Solomon Islands | 11600 | 2010 | 21100 |
| **SLE** | Sierra Leone | 31200 | 0 | 72600 |
| **SLV** | El Salvador | 111000 | 19400 | 203000 |
| **SMR** | San Marino | 0 | 0 | 0 |
| **SOM** | Somalia | 0 | 0 | 0 |
| **SPM** | Saint Pierre and Miquelon | 0 | 0 | 0 |
| **SRB** | Serbia | 319000 | 55500 | 583000 |
| **STP** | Sao Tome and Principe | 3710 | 646 | 6780 |
| **SUR** | Suriname | 10000 | 1750 | 18300 |
| **SVK** | Slovakia | 104000 | 28900 | 178000 |
| **SVN** | Slovenia | 36700 | 10200 | 63100 |
| **SWE** | Sweden | 116000 | 32200 | 199000 |
| **SWZ** | Eswatini | 19800 | 3450 | 36200 |
| **SXM** | Sint Maarten (Dutch part) | 458 | 128 | 788 |
| **SYC** | Seychelles | 1100 | 306 | 1890 |
| **SYR** | Syrian Arab Republic | 0 | 0 | 0 |
| **TCA** | Turks and Caicos Islands (the) | 429 | 120 | 739 |
| **TCD** | Chad | 63600 | 0 | 148000 |
| **TGO** | Togo | 32300 | 0 | 75100 |
| **THA** | Thailand | 824000 | 207000 | 1440000 |
| **TJK** | Tajikistan | 37200 | 0 | 86600 |
| **TKM** | Turkmenistan | 103000 | 17800 | 187000 |
| **TLS** | Timor-Leste | 22300 | 3880 | 40800 |
| **TON** | Tonga | 1800 | 314 | 3290 |
| **TTO** | Trinidad and Tobago | 15700 | 4370 | 27000 |
| **TUN** | Tunisia | 182000 | 29900 | 333000 |
| **TUR** | Turkey | 2000000 | 478000 | 3530000 |
| **TUV** | Tuvalu | 201 | 35 | 367 |
| **TWN** | Taiwan | 0 | 0 | 0 |
| **TZA** | Tanzania, United Republic of | 1290000 | 314000 | 2270000 |
| **UGA** | Uganda | 210000 | 0 | 488000 |
| **UKR** | Ukraine | 2840000 | 493000 | 5180000 |
| **UMI** | United States Minor Outlying Islands (the) | 0 | 0 | 0 |
| **URY** | Uruguay | 38900 | 10800 | 67000 |
| **USA** | United States | 2780000 | 718000 | 4850000 |
| **UZB** | Uzbekistan | 580000 | 101000 | 1060000 |
| **VAT** | Holy See (the) | 0 | 0 | 0 |
| **VCT** | Saint Vincent and the Grenadines | 1910 | 332 | 3490 |
| **VEN** | Venezuela (Bolivarian Republic of) | 0 | 0 | 0 |
| **VGB** | Virgin Islands (British) | 0 | 0 | 0 |
| **VIR** | Virgin Islands (U.S.) | 0 | 0 | 0 |
| **VNM** | Vietnam | 2180000 | 705000 | 3660000 |
| **VUT** | Vanuatu | 5180 | 900 | 9450 |
| **WLF** | Wallis and Futuna | 0 | 0 | 0 |
| **WSM** | Samoa | 3400 | 592 | 6210 |
| **YEM** | Yemen | 116000 | 0 | 271000 |
| **ZAF** | South Africa | 736000 | 151000 | 1320000 |
| **ZMB** | Zambia | 160000 | 27800 | 292000 |
| **ZWE** | Zimbabwe | 131000 | 22800 | 240000 |

**Supplementary References**

1. Page MJ, McKenzie JE, Bossuyt PM, Boutron I, Hoffmann TC, Mulrow CD, et al. The PRISMA 2020 statement: an updated guideline for reporting systematic reviews. BMJ. 2021 Mar 29;372:n71.
2. Tabelat e raportimit për intervalet kohore: 3 muaj, 9 muaj dhe vjetore si dhe relacionet përkatëse. [Internet]. Ministria e Shëndetësisë. [cited 2021 Dec 8]. Available from: <https://shendetesia.gov.al/tabelat-e-raportimit-per-intervalet-kohore-3-muaj-9-muaj-dhe-vjetore-si-dhe-relacionet-perkatese/>
3. OECD. Health at a Glance: Latin America and the Caribbean 2020 [Internet]. Paris: Organisation for Economic Co-operation and Development; 2020 [cited 2021 Dec 8]. Available from: <https://www.oecd-ilibrary.org/social-issues-migration-health/health-at-a-glance-latin-america-and-the-caribbean-2020_6089164f-en>
4. Admitted patients [Internet]. Australian Institute of Health and Welfare. [cited 2021 Dec 8]. Available from: <https://www.aihw.gov.au/reports-data/myhospitals/sectors/admitted-patients>
5. Burgess K, Australian Institute of Health and Welfare. Admitted patient care 2013-14: Australian hospital statistics. 2015.
6. Admitted patient care 2017–18, At a glance [Internet]. Australian Institute of Health and Welfare. [cited 2021 Dec 8]. Available from: <https://www.aihw.gov.au/reports/hospitals/admitted-patient-care-2017-18/contents/at-a-glance>
7. Health care use - Hospital discharge rates - OECD Data [Internet]. [cited 2021 Dec 8]. Available from: <https://data.oecd.org/healthcare/hospital-discharge-rates.htm>
8. OECD. Society at a Glance: Asia/Pacific 2019 [Internet]. OECD; 2019 [cited 2021 Dec 8]. (Society at a Glance: Asia/Pacific). Available from: <https://www.oecd-ilibrary.org/social-issues-migration-health/society-at-a-glance-asia-pacific-2019_soc_aag-2019-en>
9. Health Summary Statistics 2017.pdf [Internet]. [cited 2021 Dec 8]. Available from: <https://www.moh.gov.bh/Content/Files/Publications/statistics/HS2017/PDF/Chapters/Health%20Summary%20Statistics%202017.pdf>
10. Health summary statistics_2014.pdf [Internet]. [cited 2021 Dec 8]. Available from: <https://www.moh.gov.bh/Content/Files/Publications/statistics/HS2014/PDF/Chapters/health%20summary%20statistics_2014.pdf>
11. WHO European health information at your fingertips. [Internet]. [cited 2021 Dec 8]. Available from: <https://gateway.euro.who.int/en/indicators/hfa_534-6010-inpatient-care-discharges-per-100/visualizations/#id=19629&tab=graph>
12. Reportes Dinámicos [Internet]. [cited 2021 Dec 8]. Available from: <https://estadisticas.minsalud.gob.bo/Default.aspx>
13. Ministry of Health, Royal Government of Bhutan, Thimphu, Bhutan [Internet]. [cited 2021 Dec 8]. Available from: <https://www.moh.gov.bt/about/program-profiles/national-suicide-prevention-program/plans-orders-activities/reports/annual-health-bulletin/>
14. Access Data and Reports | CIHI [Internet]. [cited 2021 Dec 8]. Available from: <https://www.cihi.ca/en/access-data-reports/results?query=inpatient+hospitalizations+volume&Search+Submit=>
15. Statistik B für. Krankenhäuser - 1998-2019 | Tabelle [Internet]. Bundesamt für Statistik. 2020 [cited 2021 Dec 8]. Available from: <https://www.bfs.admin.ch/bfs/de/home/statistiken/kataloge-datenbanken/tabellen.assetdetail.14777227.html>
16. National Bureau of Statistics of China >> Annual Data [Internet]. [cited 2021 Dec 8]. Available from: <http://www.stats.gov.cn/english/Statisticaldata/AnnualData/>
17. 2019年我国卫生健康事业发展统计公报 [Internet]. [cited 2021 Dec 8]. Available from: <http://www.nhc.gov.cn/guihuaxxs/s10748/202006/ebfe31f24cc145b198dd730603ec4442.shtml>
18. Stasse S, Vita D, Kimfuta J, da Silveira VC, Bossyns P, Criel B. Improving financial access to health care in the Kisantu district in the Democratic Republic of Congo: acting upon complexity. Glob Health Action. 2015 Jan 5;8:10.3402/gha.v8.25480.
19. Gaitan CM, Iglesias EB, Molina WB, Morales GE. Sistema de Evaluacio´n y Caliﬁcacio´n de Actores: Ranking de Satisfaccio´n EPS 2018. :59.
20. Relatório Estatístico [Internet]. [cited 2021 Dec 8]. Available from: <https://www.minsaude.gov.cv/index.php/documentosite/-1>
21. Statistics | Eurostat [Internet]. [cited 2021 Dec 8]. Available from: <https://ec.europa.eu/eurostat/databrowser/view/hlth_co_disch2/default/table?lang=en>
22. Situation Demographique [Internet]. [cited 2021 Dec 8]. Available from: <http://www.sante.gov.dz/images/pdf/population/situation-demographique-2014.pdf>
23. Presentacion ECEH_2019.pdf [Internet]. [cited 2021 Dec 8]. Available from: <https://www.ecuadorencifras.gob.ec/documentos/web-inec/Estadisticas_Sociales/Camas_Egresos_Hospitalarios/Cam_Egre_Hos_2019/Presentacion%20ECEH_2019.pdf>
24. [Internet]. [cited 2021 Dec 8]. Available from: http://www.mohp.gov.eg/default.aspx
25. NATIONAL HEALTH ACCOUNT(NHA V).pdf [Internet]. [cited 2021 Dec 8]. Available from: <https://ehia.gov.et/sites/default/files/Resources/NATIONAL%20HEALTH%20ACCOUNT(NHA%20V).pdf>
26. Health-financing-in-the-Republic-of-Gabon.pdf [Internet]. [cited 2021 Dec 8]. Available from: <https://documents1.worldbank.org/curated/en/875521468252026617/pdf/Health-financing-in-the-Republic-of-Gabon.pdf>
27. WHO European health information at your fingertips. [Internet]. [cited 2021 Dec 8]. Available from: <https://gateway.euro.who.int/en/indicators/hfa_535-6011-number-of-all-hospital-discharges/visualizations/#id=19630&tab=table>
28. Badan Pusat Statistik [Internet]. [cited 2021 Dec 8]. Available from: <https://www.bps.go.id/statictable/2009/03/10/1559/indikator-kesehatan-1995-2020.html>
29. KI_Health_75th_Final.pdf [Internet]. [cited 2021 Dec 8]. Available from: <http://mospi.nic.in/sites/default/files/publication_reports/KI_Health_75th_Final.pdf>
30. nss_71st_ki_health_30june15.pdf [Internet]. [cited 2021 Dec 8]. Available from: <http://mospi.nic.in/sites/default/files/publication_reports/nss_71st_ki_health_30june15.pdf>
31. Beiranvand S, Saki M, Behzadifar M, Bakhtiari A, Behzadifar M, Keshvari M, et al. The effect of the Iranian health transformation plan on hospitalization rate: insights from an interrupted time series analysis. BMC Health Services Research. 2020 Apr 19;20(1):327.
32. وزارة الصحة العراقية. [Internet]. [cited 2021 Dec 8]. Available from: <https://moh.gov.iq/>
33. Jamaica Open Data [Internet]. [cited 2021 Dec 8]. Available from: <https://data.gov.jm/dataset/2017-national-health-statistics/resource/e988c2e2-9803-421e-904a-3906ffd5a0f9#{}>
34. Health2019.pdf [Internet]. [cited 2021 Dec 8]. Available from: <http://dosweb.dos.gov.jo/wp-content/uploads/2020/06/Health2019.pdf>
35. yearbook_2014.pdf [Internet]. [cited 2021 Dec 8]. Available from: <http://dosweb.dos.gov.jo/wp-content/uploads/2018/02/yearbook_2014.pdf>
36. Kenya National Data Archive (KeNADA) An Online Microdata Catalog [Internet]. [cited 2021 Dec 8]. Available from: <https://statistics.knbs.or.ke/nada/index.php/home>
37. របាយការណ៍​វឌ្ឍនភាព – Ministry of Health [Internet]. [cited 2021 Dec 8]. Available from: <http://moh.gov.kh/%e1%9e%9a%e1%9e%94%e1%9e%b6%e1%9e%99%e1%9e%80%e1%9e%b6%e1%9e%9a%e1%9e%8e%e1%9f%8d%e2%80%8b%e1%9e%9c%e1%9e%8c%e1%9f%92%e1%9e%8d%e1%9e%93%e1%9e%97%e1%9e%b6%e1%9e%96/>
38. service_availability_and_readiness_assessment_final_12-03-2018.pdf [Internet]. [cited 2021 Dec 8]. Available from: <https://www.humanitarianresponse.info/sites/www.humanitarianresponse.info/files/assessments/service_availability_and_readiness_assessment_final_12-03-2018.pdf>
39. sante en chiffres 2017.pdf [Internet]. [cited 2021 Dec 8]. Available from: <https://www.sante.gov.ma/Publications/Etudes_enquete/Documents/2021/sante%20en%20chiffres%202017.pdf>
40. Myanmar: number of hospital admissions [Internet]. Statista. [cited 2021 Dec 8]. Available from: <https://www.statista.com/statistics/1060134/myanmar-number-hospital-admissions/>
41. 2019-eruul_mendin_uzuulelt_MU_mail.indd_2020_______7___21final.pdf [Internet]. [cited 2021 Dec 8]. Available from: <http://hdc.gov.mn/media/uploads/2020-08/2019-eruul_mendin_uzuulelt_MU_mail.indd_2020_______7___21final.pdf>
42. Portal Rasmi Kementerian Kesihatan Malaysia [Internet]. [cited 2021 Dec 8]. Available from: <https://www.moh.gov.my/index.php/pages/view/58?mid=19>
43. OECD, Organization WH. Health at a Glance: Asia/Pacific 2014 Measuring Progress towards Universal Health Coverage: Measuring Progress towards Universal Health Coverage. OECD Publishing; 2014. 120 p.
44. Anuarios Estadísticos | Ministerio de Salud de la República de Panamá [Internet]. [cited 2021 Dec 8]. Available from: <http://www.minsa.gob.pa/informacion-salud/anuarios-estadisticos>
45. cap06.pdf [Internet]. [cited 2021 Dec 8]. Available from: <https://www.inei.gob.pe/media/MenuRecursivo/publicaciones_digitales/Est/Lib1635/cap06/cap06.pdf>
46. Dirección de Salud Bucodental [Internet]. [cited 2021 Dec 8]. Available from: [https://portal.mspbs.gov.py/#](https://portal.mspbs.gov.py/)
47. Health & Welfare [Internet]. [cited 2021 Dec 8]. Available from: <http://www.nso.go.th/sites/2014en/Pages/Statistical%20Themes/Population-Society/Social%20Security/Health--Welfare.aspx>
48. 統計處. Statistics of Medical Care Institution’s Status & Hospital Utilization 2019 [Internet]. 統計處. 統計處; 2020 [cited 2021 Dec 8]. Available from: <https://www.mohw.gov.tw/cp-4932-54834-2.html>
49. Opendi and Moriku - Hon. Dr. Jane Ruth Aceng Minister of Health.pdf [Internet]. [cited 2021 Dec 8]. Available from: <http://library.health.go.ug/sites/default/files/resources/AHSPR%202018_19%20FY%20Final%20copy.pdf>
50. AHA Stats [Internet]. [cited 2021 Dec 8]. Available from: <https://guide.prod.iam.aha.org/stats/historical-trends-utilization>
51. OECD. Hospital discharges [Internet]. Paris: OECD; 2015 Nov [cited 2021 Dec 8] p. 106–7. Available from: <https://www.oecd-ilibrary.org/social-issues-migration-health/health-at-a-glance-2015/hospital-discharges_health_glance-2015-33-en>
52. ZamStats - Publications - Other Institutions [Internet]. [cited 2021 Dec 8]. Available from: <https://www.zamstats.gov.zm/index.php/publications/category/21-other-institutions>
53. Zeng W, Lannes L, Mutasa R. Utilization of Health Care and Burden of Out-of-Pocket Health Expenditure in Zimbabwe: Results from a National Household Survey. Health Systems & Reform. 2018 Oct 2;4(4):300–12
